# *Enterococcus hirae* biofilm formation on hospital material surfaces and effect of new biocides

**DOI:** 10.1186/s12199-017-0670-3

**Published:** 2017-08-02

**Authors:** Silvia Di Lodovico, Valentina Cataldi, Emanuela Di Campli, Elisabetta Ancarani, Luigina Cellini, Mara Di Giulio

**Affiliations:** Department of Pharmacy, University “G. d’Annunzio” Chieti-Pescara, Chieti, Italy

**Keywords:** Biocidal products, Biofilm, *Enterococcus hirae*, Hospital-acquired infections (HAIs), Sanitation

## Abstract

**Background:**

Nowadays, the bacterial contamination in the hospital environment is of particular concern because the hospital-acquired infections (HAIs), also known as nosocomial infections, are responsible for significant morbidity and mortality. This work evaluated the capability of *Enterococcus hirae* to form biofilm on different surfaces and the action of two biocides on the produced biofilms.

**Methods:**

The biofilm formation of *E. hirae* ATCC 10541 was studied on polystyrene and stainless steel surfaces through the biomass quantification and the cell viability at 20 and 37 °C. The effect of LH IDROXI FAST and LH ENZYCLEAN SPRAY biocides on biomasses was expressed as percentage of biofilm reduction. *E. hirae* at 20 and 37 °C produced more biofilm on the stainless steel in respect to the polystyrene surface. The amount of viable cells was greater at 20 °C than with 37 °C on the two analyzed surfaces. Biocides revealed a good anti-biofilm activity with the most effect for LH ENZYCLEAN SPRAY on polystyrene and stainless steel at 37 °C with a maximum biofilm reduction of 85.72 and 86.37%, respectively.

**Results:**

*E. hirae* is a moderate biofilm producer depending on surface material and temperature, and the analyzed biocides express a remarkable antibiofilm action.

**Conclusion:**

The capability of *E. hirae* to form biofilm can be associated with its increasing incidence in hospital-acquired infections, and the adoption of suitable disinfectants is strongly recommended.

**Electronic supplementary material:**

The online version of this article (doi:10.1186/s12199-017-0670-3) contains supplementary material, which is available to authorized users.

## Background

Nowadays, the bacterial contamination in the hospital environment is of particular concern because the hospital-acquired infections (HAIs), also known as nosocomial infections, are responsible for significant morbidity and mortality [1]. The European Centre for Disease Prevention and Control (ECDC) estimated that, in the EU, each year, about 4, 100,000 patients acquired a healthcare-associated infection, resulting in 110, 000 deaths [2].

According to ECDC, HAIs are infections contracted in the healthcare setting (e.g., inpatient hospital admission or same-day surgery) that can originate from different sources such as external environment, infected patients, healthcare staff that may be infected, contaminated items (food, water, medications, devices, and equipment), or droplets containing microbes [3, 4].

It is well known that bacteria, exposed to various stresses (e.g., antibiotics, nutrient limitation, non-permissive temperature), can express the ability to form multicellular organizations as a network of cell-to-cell interactions attached to each other and/or to surfaces, called biofilms that permit survival in adverse environments [5], and that are difficult to treat, resulting in an enormous impact on healthcare [6–9].

Therefore, the capability of bacteria to grow on biofilm mode surfaces is in another aspect to consider in the clinical contamination.

Actually, equipment for sanitation and hand-touch surfaces sanitizing/sterilization together with hand washing of visitors to patients, and all medical personnel are the most effective ways to contrast nosocomial infections [10]. Consequently, hospital disinfection policies play an mportant role in the control of HAIs [11, 12]. “Biocidal products are those that are intended to destroy, render harmless, prevent the action of, or otherwise exert a controlling effect on any harmful organism by chemical or biological means; examples include disinfectants, preservatives, antiseptics, pesticides, herbicides, fungicides and insecticides” (Biocides Directive 98/8/EC 1998) [13]. They are used widely for the disinfection of surfaces and equipment, and for sterilization of medical devices.

Enterococci, previously included among gut commensals of humans and animals, in the last years, have acquired the role of common nosocomial pathogens (the third leading source of nosocomial infection), causing urinary tract infections, endocarditis, peritonitis, and bacteremia [14]. Among these, *Enterococcus hirae*, proposed as a new test germ within the framework of the procedures for the European standardization of chemical and antiseptic agents for evaluation and validation of disinfectant products (EN14561:2006) [15], has been recently described as an emergent nosocomial pathogen in HAIs [16, 17]. Despite, human *E. hirae* infection is extended to be 1–3% of the *Enterococcus* spp. infections detected in clinical practice [18], and an emerging role of source of serious illness can be attributed to this microorganism responsible of endocarditis, acute pancreatitis, pyelonephritis, and septic shock [18–23]. In sight of this and taking into account the role that the biofilms have in hand-touch surface-associated nosocomial infection, our aim was to characterize the biofilm formation of *E. hirae* on polystyrene and stainless steel surfaces, and to investigate the antibiofilm action of two biocides, to be used in the disinfection of surgical and the medical device.

## Methods

### Strain culture condition

The reference strain *E. hirae* ATCC 10541 was used for this study. To recover the strain, a loop of cell was picked up from the strain stored at −80 °C and cultured in Tryptic Soy Broth (TSB, Oxoid, Milan, Italy) plus 1% *v*/*v* of glucose (Sigma Aldrich, Milan, Italy) (TSBG) at 37 °C overnight under aerobic condition. After incubation, the broth culture was diluted 1:10 in the same medium and refreshed for 2 h at 37 °C in shaking thermostat water bath (160 rpm). Finally, the culture was adjusted in spectrophotometer (Eppendorf, Milan, Italy) to optical density OD_600_ = 0.12 corresponding to 0.5 Mcfarland [24]. This broth culture standardized was used for the experiments.

### In vitro biofilm formation and biomass quantification

The biofilm formation of *E. hirae* was evaluated on polystyrene and stainless steel, two surface materials widely used in the hospital, at two different incubation temperatures, 20 and 37 °C [25]. For the analysis on polystyrene surface, the standardized broth culture (200 μL) was inoculated on flat-bottomed 96-well polystyrene microtiter plates, and incubated at 20 and 37 °C for 48 h. After incubation, the planktonic cells were removed from each well and biofilms and the respective negative control (TSBG without bacteria) were rinsed with sterile water, fixed by air drying, and stained with Crystal Violet 0.1% (Sigma Aldrich, Milan, Italy) for 1 min. The stained biofilms were washed with sterile water and eluted with ethanol for reading.

For analysis on stainless steel surface, sample sheets (0.5 mm thickness) of stainless steel, obtained locally, were divided into small coupons (1 cm × 1.5 cm) and used as surfaces for biofilm growth. Prior to testing, stainless steel coupons were washed with detergent, rinsed with distilled water, immersed in 70% ethanol, rinsed again with distilled water, and finally sterilized [26].

Two microliters of *E. hirae* ATCC 10541 standardized broth culture were used to cover totally sterile stainless steel coupons, placed into Petri dishes (3.5 cm of diameter). Petri dishes were incubated aerobically at 20 and 37 °C for 48 h. After incubation, the planktonic bacteria were removed from Petri dishes and biofilms were washed with sterile water, dried as previously described [27] and stained with Crystal Violet 0.1% for 1 min, washed with sterile water, and eluted with ethanol. Two hundred microliters of eluted solution were read by using a microplate reader (SAFAS, Munich, Germany) with an absorbance of 595 nm.

Three independent experiments in triplicate, for each temperature and material surface, were performed.

Afterwards, using the OD_595_ measurements of biofilms formed, *E. hirae* ATCC 10541 was classified as strong, moderate, or weak biofilm producer according to Stepanovic et al. [28], as follows: OD ≤ O.D.c = no biofilm producer, O.D.c < OD ≤ (2× O.D.c) = weak biofilm producer, (2× O.D.c) < OD ≤ (4× O.D.c) = moderate biofilm producer and (4× O.D.c) ≤ OD = strong biofilm producer. The cut-off O.D.c was defined as three standard deviations above the mean OD of the negative control.

### Action of biocides in the removal of biofilms formed on polystyrene and stainless steel

The two biocides were provided by Lombarda H S.r.l. (Albairate, Milan, Italy). The chemical characteristics are showed in Table 1, and they were used at concentration recommended by the biocide manufacture. The biofilms of *E. hirae* ATCC 10541 on polystyrene, and stainless steel were performed under the same conditions described above. After 48 h of formation at 20 and 37 °C, the planktonic cells were removed and the biofilms formed on materials were treated with biocides for 60 min. All experiments included controls (with biocides) and TSBG (without bacteria). After incubation, the treated biofilms were washed, stained with Crystal Violet 0.1% for 1 min, washed with sterile water, eluted with ethanol, and read as described above.

**Table 1:** Chemical characteristics of the two biocides used

Biocides	Chemical composition (%, *w*/*w*)
LH IDROXI FAST	Ethyl alcohol 9%, hydrogen peroxide 5%, preservatives, purified water to 100 mL
LH ENZYCLEAN SPRAY	Benzalkonium chloride 0.6%, didecilammonio chloride 0.6%, non-ionic surfactant 3.2%, protease 0.2%, 0.1% amylase, lipase 0.1%, isopropilico 2% alcohol, co-formulants, and purified water to 100 mL

Three independent experiments in triplicate, for each temperature and material surface tested, were performed.

For control, the antibiofilm action of the two biocides was also evaluated against a strong biofilm producer microorganism. To do this, the *Staphylococcus aureus* ATCC 6538 biofilm was performed at 37 °C for 24 h on polystyrene surface and treated with biocides as described above.

The antibiofilm action of biocides was expressed as percentage of reduction of biofilm biomass in respect to biofilm formed on each material tested with biocides.

### Cell viability assay

For the evaluation of cells viability in produced biofilms, a BacLight LIVE/DEAD Viability Kit (Molecular Probes, Invitrogen detection technologies, USA) was used. SYTO 9 stains viable cells with a green fluorescent signal, and propidium iodide stains cells with impaired membrane activity red.

Three independent experiments on Petri dishes (3.5 cm of diameter) and on stainless steel coupons for each temperature tested were performed in triplicate as described above.

After 48 h of incubation, the planktonic cells were removed both from each Petri dish and stainless steel coupon; the sessile bacterial populations on the material surfaces were washed with PBS and stained as indicated by manufacturer. The images observed at fluorescent Leica 4000 DM microscopy (Leica Microsystems, Milan, Italy) were recorded at an emission wavelength of 500 nm for SYTO 9 (green fluorescence) and of 635 nm for propidium iodide (red fluorescence) and more fields of view randomly were examined.

### Concanavalin A assay

To visualize the extracellular polymeric substance (EPS) matrix of the produced biofilms, rhodamine-labeled Concanavalin A (rhodamine-conA) (Vector Laboratories, Burlingame, CA, USA), which specifically binds to d-(+)-glucose and d-(+)-mannose groups on EPS, was used. Three independent experiments on Petri dishes (3.5 cm of diameter) and on stainless steel coupons for each temperature tested were performed in triplicate as described above. After 48 h of incubation, the planktonic cells were removed both from each Petri dishes and stainless steel coupons; the sessile bacterial populations were washed with PBS and stained with the rhodamine-conA (10 μg/mL). After a 30 min incubation in the dark at room temperature, the excess staining solution was removed, rinsed with PBS, and examined under fluorescence Leica 4000 DM microscopy. Images were recorded at an excitation of 514 nm and an emission wavelength of 600 ± 50 nm.

### Statistical analyses

All data were expressed as the mean ± standard deviation (SD) of three independent experiments in triplicate. The statistical significance of the obtained differences was evaluated using *t* student test.

Values of *p* < 0.05 were considered statistically significant.

## Results

**Fig. 1:**
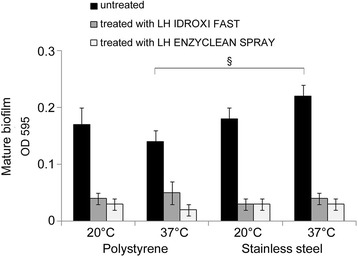
The ability of *E. hirae* ATCC 10541 to form biofilm on polystyrene and stainless steel, at 20 and 37 °C and in vitro antibiofilm action of disinfectants LH IDROXI FAST and LH ENZYCLEAN SPRAY tested at the same conditions. The untreated and treated biofilms were analyzed for the biomass production, after 48 h of incubation, through Cristal Violet staining method. The results were expressed as average of OD595 values of three experiments (mean value ± SD). Symbol represents result statistically significant (*p* <0.05). In particular, § represents the statistically significant difference in the values observed between polystyrene and stainless steel at 37 °C

The biofilms produced by *E. hirae* ATCC 10541 on polystyrene and on stainless steel, at two different temperatures are shown in Fig. 1. The microorganism, at both 20 and 37 °C, produced more biofilm on stainless steel (OD_595_ = 0.18 ± 0.02 and OD_595_ = 0.22 ± 0.02, respectively) than polystyrene (OD_595_ = 0.17 ± 0.03 and OD_595_ = 0.14 ± 0.02, respectively). The difference in the biofilm values observed on polystyrene and stainless steel at 37 °C was statistically significant (*p* = 0.002). The capability of strain to form biofilm on polystyrene and stainless steel surfaces was quantified according to Stepanovic et al. classification. On the bases of the obtained data, the bacterium was defined a weak biofilm producer on polystyrene surface at the two tested temperatures and on stainless steel surface at 20 °C, whereas was classified as a moderate biofilm producer on stainless steel at 37 °C.

**Fig. 2:**
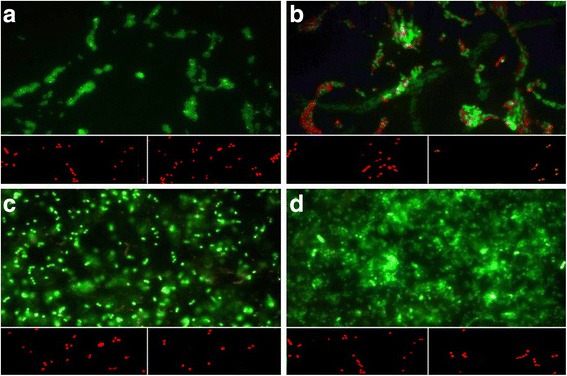
Representative images of the in vitro mature biofilms on polystyrene at 20 °C (**a**) and 37 °C (**b**) and on stainless steel coupon at 20 °C (**c**) and 37 °C (**d**) of *E. hirae* ATCC 10541, and the effect of disinfectants LH IDROXI FAST (*inserts on the left*) and LH ENZYCLEAN SPRAY (*inserts on the right*) tested at the same conditions. Biofilms were cultured for 48 h, stained with live/dead reagents, and visualized with the optical microscope fluorescence. Sessile population in biofilms stained in red (propidium iodide) expresses a compromised membrane integrity (damaged), whereas green stained bacteria (SYTO 9) remained viable. Original magnification ×1000

Figure 2 shows the cellular viability of mature biofilms formed on polystyrene at 20 °C (a) and 37 °C (b) and on stainless steel coupon at 20 °C (c) and 37 °C (d) and the effect of disinfectants LH IDROXI FAST (inserts on the left) and LH ENZYCLEAN SPRAY (inserts on the right) tested at the same conditions. At each tested temperature, bacteria were more adherents on stainless steel than polystyrene surface (*p <* 0.05). On polystyrene surface, the amount of green viable cells was greater at 20 than 37 °C whereas, on stainless steel coupon, this difference was not detected. Both biocides displayed a full killing effect in each detected condition with red dead cells.

**Fig. 3:**
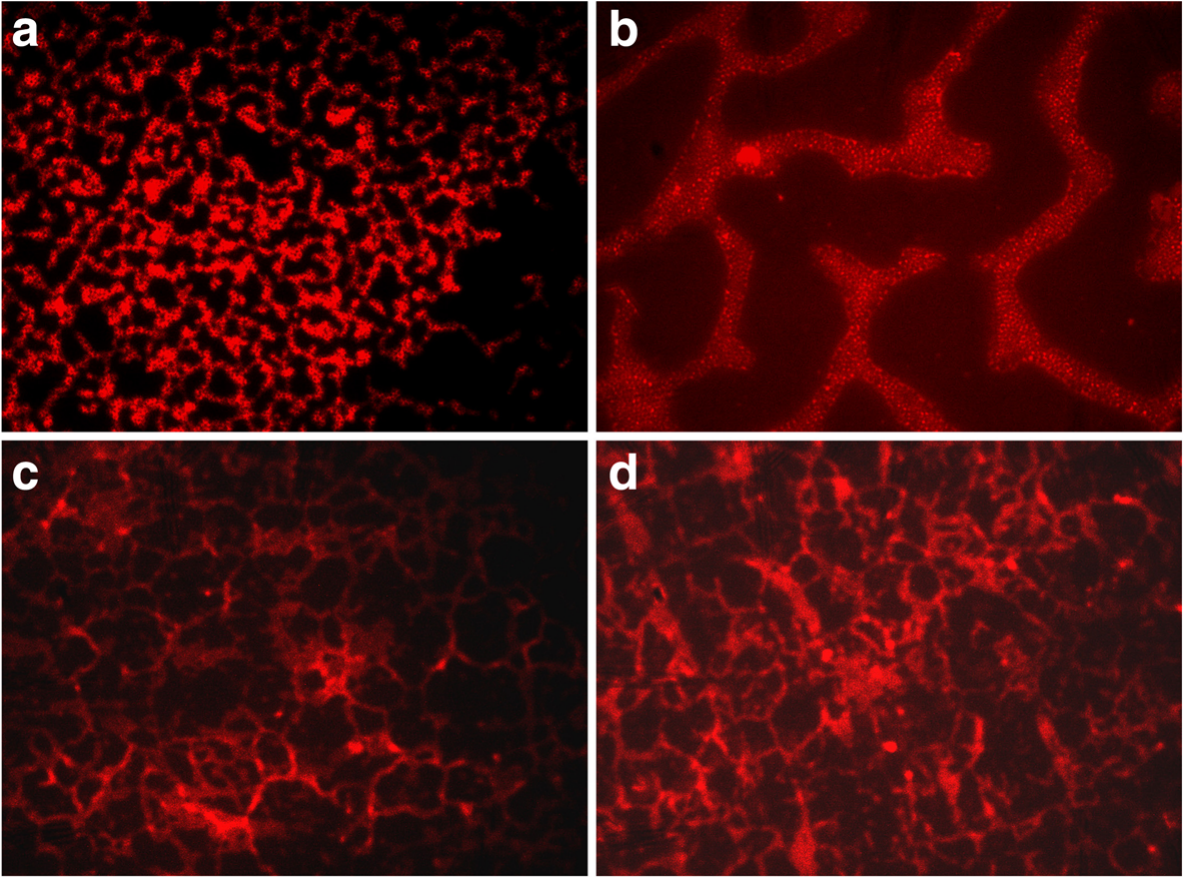
Optical microscope fluorescence representative images of *E. hirae* ATCC 10541 biofilms at 48 h on polystyrene at 20 °C (**a**) and 37 °C (**b**) on stainless steel coupon at 20 °C (**c**) and 37 °C (**d**) by Con-A staining for detecting α glucans in matrix formation. Original magnification ×1000

Moreover, we also evaluated the presence of the biofilm exopolysaccharide component formed on two material surfaces through the Concanavalin A assay. As shown in Fig. 3, a major extracellular matrix production was performed on stainless steel coupon at both temperatures in respect to the other tested surface (*p* < 0.05).

The results regarding the biomass production by *E. hirae* ATCC 10541 on material, following treatment with two biocides are shown in Fig. 1. LH IDROXI FAST and LH ENZYCLEAN SPRAY, used at undiluted concentration, exhibited a massive decrease of biofilm biomass on polystyrene and stainless steel at both temperatures tested that was significant in respect to untreated samples (Table 2). In particular, after 60 min of contact, LH ENZYCLEAN SPRAY displayed the most antibiofilm activity on polystyrene and stainless steel at 37 °C with a biofilm reduction of 85.72 and 86.37%, respectively. Biocides, were also effective in their antibiofilm activity when used against the strong biofilm producer. After 60 min of contact, LH IDROXI FAST and LH ENZYCLEAN SPRAY showed a percentage of *S. aureus* ATCC 6538 biofilm reduction, in respect to the untreated control, of 60.4 and 69.8, respectively, even exerting a killing effect (see Additional file 1: Figure S1).

**Table 2:** In vitro antibiofilm action of biocides on *E. hirae* ATCC 10541 grown on polystyrene and stainless steel, expressed as relative reduction (%) of biofilm biomass in respect to the untreated sample (control), after 60 min of contact at 20 and 37 °C. The biocides were used at concentration recommended by manufacturers

	Percentage of relative biofilm reduction			
Biocides	20 °C		37 °C	
	Polystyrene (%)	Stainless steel (%)	Polystyrene (%)	Stainless steel (%)
LH IDROXI FAST	76.47	83.34	64.29	81.82
LH ENZYCLEAN SPRAY	82.36	83.34	85.72	86.37

## Discussion

In this study, we first evaluated the biofilm formation by *E. hirae* ATCC 10541 on two surface materials, commonly used in the hospital setting, at 20 and 37 °C. We chose polystyrene, a material that covers more than 25% of all plastic surfaces, both in medical applications and in common fittings present in hospital rooms and stainless steel included in surgical instruments and in orthopedic, craniofacial, and cardiovascular implant devices or on door handles in surgery rooms.

Our results reveal that the capability of *E. hirae* to form biofilm was greater on stainless steel than polystyrene, at each temperature tested. It is widely known that the bacterial adhesion to surfaces, that results from interplay of forces, including Van der Waals, electrostatic and hydrophobic interactions, is affected by various factors. In particular, the properties of surfaces (chemical composition, charge, hydrophobicity, roughness and texture) are strongly related with the capability of bacteria to form biofilm. As confirmed by our data, rough and hydrophobic surfaces as stainless steel, favor the bacterial adhesion in respect to smooth surfaces as well as polystyrene. Adding to this, we also evaluated the effect of the temperature on the bacterial adhesion on surface. In particular, for each material, we considered two temperatures, 20 °C, a typical temperature present in the surgery room and 37 °C. Although Enterococci are able to grow in a wide range of temperatures (5-50 °C), 37 °C represents the best condition, increasing both the cell number and the biomass, resulting in a higher degree of initial adhesion to surfaces [29, 30]. However, the capability of adherence at the two different temperatures underlines the microorganism resilience and, consequently, its easy nosocomial spread as also noticed for other enterococcal species [31].

From our study, on polystyrene surface, the produced biofilm was higher at 20 °C in respect to 37 °C; on the contrary, on stainless steel surface, 37 °C was the temperature that favored the major adhesion, confirming the correct use in the clinical settings of stainless steel at low temperature environments such as surgery rooms.

In addition, Lleo et al. [32] studied the persistence of *Enterococcus* spp. including *E. hirae* on polystyrene medical devices, defining the biofilm formation as a strategy for the beginning and maintaining of infection nosocomial diseases.

The formed biofilms on stainless steel at 20 and 37 °C, analyzed by cell viability assay, displayed a copious presence of green bio-volume. In the case of polystyrene at 37 °C, the less amount of green cells, also associated with damaged red cells, reflected the outcome of biomass biofilm analyses. All images related to Concanavalin A assay reflect the situations described above, and in addition, showing the considerable EPS matrix production at 20 °C on polystyrene and 37 °C stainless steel.

Microbial biofilms are well known for their high resistance to biocide treatment, making most of the device-related infections difficult to eradicate. Due to the important role that the biocides have in the control of nosocomial infection, we tested two new commercial disinfectants on *E. hirae* sessile population grown on the same materials. This microorganism, included among the bacterial species necessary for the validation of nosocomial biocides, represents an emergent opportunistic pathogen to be addressed in terms of sanitation control. Both biocides showed an important antibiofilm activity against formed biofilms by bacteria in each condition. In particular, LH ENZYCLEAN SPRAY that contains benzalkonium chloride as active ingredients in the formulation, displayed a stronger antibiofilm effect than LH IDROXI FAST, probably for its capability to interact with the whole cell microbial body. Moreover, as reported for others *Enterococcus* spp., the wide antimicrobial resistance often detected for these species was not correlated to biocide tolerance [33]. This antibiofilm effect was also obtained by using *S. aureus* ATCC 6538, a strong biofilm producer, confirming the capability of the biocides to be effective also against well-structured sessile population. Overall, this important strong antibiofilm effect together with the surface compatible [11], underline their appropriate use in health care.

## Conclusion

In conclusion, *E. hirae* is capable to form biofilm and this skill is dependent both on the surface material and the temperature and can be associated to its increasing incidence in HAIs. Moreover, the analyzed biocides can be suggested as suitable disinfectants to plan the success of sanitization procedures against *E. hirae* biofilms.

## Additional file

Additional file 1: Figure S1. In vitro effect of LH IDROXI FAST and LH ENZYCLEAN SPRAY on mature biofilm of *S. aureus* ATCC 6538. Top; the untreated and treated biofilms were analyzed for the biomass production, after 48 h of incubation at 37 °C on polystyrene surface, through Cristal Violet staining method. The results were expressed as average of OD595 values of three experiments (mean value ± SD). Symbol represents result statistically significant (*p* ˂ 0.05). Down; representative images of the in vitro mature biofilms at 37 °C on polystyrene surface untreated (a) and treated with LH IDROXI FAST (b) and LH ENZYCLEAN SPRAY (c). Biofilms were cultured for 48 h, stained with live/dead reagents, and visualized with the optical microscope fluorescence. Sessile population in biofilms stained in red (propidium iodide) expresses a compromised membrane integrity (damaged), whereas green stained bacteria (SYTO 9) remained viable. Both biocides reduced significantly *S. aureus* ATCC 6538 biomasses even exerting a killing effect. Original magnification ×1000. (ZIP 114 kb)

## Abbreviations

ATCC: American Type Culture Collection
CDC: Centers for Disease Control and Prevention
*E. faecalis*: *Enterococcus faecalis*
*E. faecium*: *Enterococcus faecium*
*E. hirae*: *Enterococcus hirae*
ECDC: European Centre for Disease Prevention and Control
EPS: Extracellular polymeric substances
EU: European Union
HAIs: Hospital-acquired infections
O.D.: Optical density
O.D.c: Cut-off optical density
PBS: Phosphate buffered saline
SD: Standard deviation
TSB: Tryptic Soy Broth
TSBG: Tryptic Soy Broth with glucose

## References

[1] Sahu MK, Siddharth B, Choudhury A, Vishnubhatla S, Singh SP, Menon R (2016). Incidence, microbiological profile of nosocomial infections, and their antibiotic resistance patterns in a high volume Cardiac Surgical Intensive Care Unit. Ann Card Anaesth.

[2] European Centre for Disease Prevention and Control (ECDPC). Annual epidemiological report 2014––antimicrobial resistance and healthcare-associated infections. Available at: https://ecdc.europa.eu/sites/portal/files/media/en/publications/Publications/antimicrobial-resistance-annual-epidemiological-report.pdf.

[3] Emily RM, Sydnor TM (2011). Hospital epidemiology and infection control in acute-care settings. Clin Microbiol Rev.

[4] Weber DJ, Anderson D, Rutala WA (2013). The role of the surface environment in healthcare-associated infections. Curr Opin Infect Dis.

[5] de la Fuente-Núñez C, Reffuveille F, Fernández L, Hancock RE (2013). Bacterial biofilm development as a multicellular adaptation: antibiotic resistance and new therapeutic strategies. Curr Opin Microbiol.

[6] Gomes LC, Silva LN, Simões M, Melo LF, Mergulhão FJ (2015). *Escherichia coli* adhesion, biofilm development and antibiotic susceptibility on biomedical materials. J Biomed Mater Res A.

[7] Abreu AC, Tavares RR, Borges A, Mergulh~ao F, Sim~oes M (2013). Current and emergent strategies for disinfection of hospital environments. J Antimicrob Chemother.

[8] Cataldi V, Di Bartolomeo S, Di Campli E, Nostro A, Cellini L, Di Giulio M (2015). In vitro activity of *Aloe vera* inner gel against micro-organisms grown in planktonic and sessile phases. Int J Immunopathol Pharmacol.

[9] Sepehr S, Rahmani-Badi A, Babaie-Naiej H, Soudi MR (2014). Unsaturated fatty acid, cis-2-decenoic acid, in combination with disinfectants or antibiotics removes pre-established biofilms formed by food-related bacteria. PLoS One.

[10] Collins AS (2008). Patient safety and quality: an evidence-based handbook for nurses. Preventing health care–associated infections.

[11] Rutala WA, Weber DJ (2013). Disinfectants used for environmental disinfection and new room decontamination technology. Am J Infect Control.

[12] Rutala WA, Weber DJ (2013). Disinfection and sterilization: an overview. Am J Infect Control.

[13] Biocides Directive 98/8/EC of the European Parliament and of the Council of 16 February 1998 concerning the placing of biocidal products on the market.

[14] Paganelli FL, Willems RJ, Leavis HL (2012). Optimizing future treatment of enterococcal infections: attacking the biofilm?. Trends Microbiol.

[15] EN 14561:2006 Chemical disinfectants and antiseptics. Quantitative carrier test for the evaluation of bactericidal activity for instruments used in the medical area. Test method and requirements (phase 2, step 2).

[16] Bourafa N, Loucif L, Boutefnouchet N, Rolain JM (2015). *Enterococcus hirae,* an unusual pathogen in humans causing urinary tract infection in a patient with benign prostatic hyperplasia: first case report in Algeria. New Microbes New Infect.

[17] Talarmin JP, Pineau S, Guillouzouic A, Boutoille D, Giraudeau C, Reynaud A (2011). Relapse of *Enterococcus hirae* prosthetic valve endocarditis. J Clin Microbiol.

[18] Pãosinho A, Azevedo T, Alves JV, Costa IA, Carvalho G, Peres SR (2016). Acute pyelonephritis with bacteremia caused by *Enterococcus hirae*: a rare infection in humans. Case Rep Infect Dis.

[19] Poyart C, Lambert T, Morand P, Abassade P, Quesne G, Baudouy Y (2002). Native valve endocarditis due to *Enterococcus hirae*. J Clin Microbiol.

[20] Vinh DC, Nichol KA, Rand F, Embil JM (2006). Native-valve bacterial endocarditis caused by *Lactococcus garvieae*. Diagn Microbiol Infect Dis.

[21] Dicpinigaitis PV, De Aguirre M, Divito J (2015). *Enterococcus hirae* bacteremia associated with acute pancreatitis and septic shock. Case Rep Infect Dis.

[22] Sim JS, Kim HS, Oh KJ, Park MS, Jung EJ, Jung YJ (2012). Spontaneous bacterial peritonitis with sepsis caused by *Enterococcus hirae*. J Korean Med Sci.

[23] Brulé N, Corvec S, Villers D, Guitton C, Bretonnière C (2013). Life-threatening bacteremia and pyonephrosis caused by *Enterococcus hirae*. Med Mal Infect.

[24] Di Giulio M, di Giacomo V, Di Campli E, Di Bartolomeo S, Zara S, Pasquantonio G (2013). Saliva improves *Streptococcus mitis* protective effect on human gingival fibroblasts in presence of 2-hydroxyethyl-methacrylate. J Mater Sci Mater Med.

[25] Baldassarri L, Cecchini R, Bertuccini L, Ammendolia MG, Iosi F, Arciola CR (2001). Enterococcus spp. produces slime and survives in rat peritoneal macrophages. Med Microbiol Immunol.

[26] Parizzi SQF, Andrade NJ, Silva AS, Soares NFFS, Silva AM (2004). Bacterial adherence to different inert surfaces evaluated by epifluorescence microscopy and plate count method. Braz Arch Biol Technol.

[27] Di Giulio M, Traini T, Sinjari B, Nostro A, Caputi S and Cellini L. *Porphyromonas gingivalis* biofilm formation in different titanium surfaces, an *in vitro* study. Clin Oral Implants Res 2015; doi: 10.1111/clr.12659. 10.1111/clr.12659 26249670

[28] Stepanovic S, Vukovic D, Dakic I, Savic B, Svabic-Vlahovic M (2000). A modified microtiter-plate test for quantification of staphylococcal biofilm formation. J Microbiol Methods.

[29] Arnold JW, Bailey GW (2000). Surface finishes on stainless steel reduce bacterial attachment and early biofilm formation: scanning electron and atomic force microscopy study. Poult Sci.

[30] Fisher K, Phillips C (2009). The ecology, epidemiology and virulence of Enterococcus. Microbiology.

[31] Van Wamel WJ, Hendrickx AP, Bonten MJ, Top J, Posthuma G, Willems RJ (2007). Growth condition-dependent Esp expression by *Enterococcus faecium* affects initial adherence and biofilm formation. Infect Immun.

[32] Lleo M, Bonato B, Tafi MC, Caburlotto G, Benedetti D, Canepari P (2007). Adhesion to medical device materials and biofilm formation capability of some species of enterococci in different physiological states. FEMS Microbiol Lett.

[33] Rizzotti L, Rossi F, Torriani S (2016). Biocide and antibiotic resistance of *Enterococcus faecalis* and *Enterococcus faecium* isolated from the swine meat chain. Food Microbiol.

