# Supplementary figures and images for: Enterococcus hirae biofilm formation on hospital material surfaces and effect of new biocides
Source: Environ Health Prev Med. 2017 Aug 2;22:63. doi: 10.1186/s12199-017-0670-3 (PMC5664585; doi:10.1186/s12199-017-0670-3)

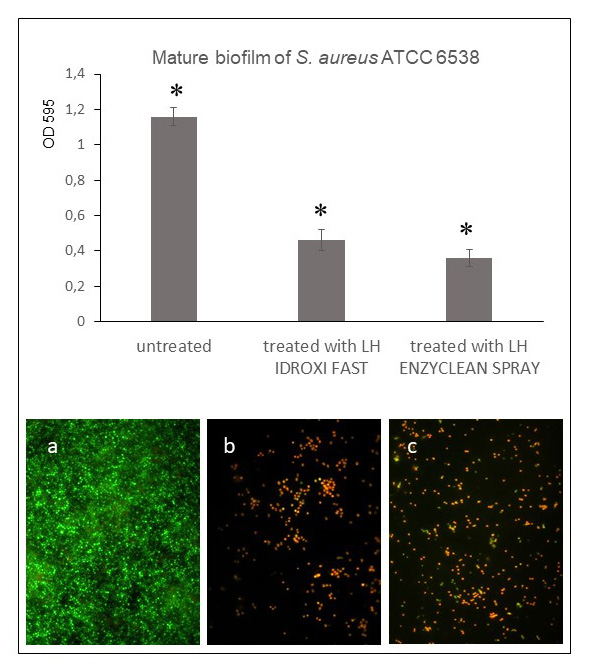

Supplement: Additional file 1: Figure S1. — In vitro effect of LH IDROXI FAST and LH ENZYCLEAN SPRAY on mature biofilm of S. aureus ATCC 6538. Top; the untreated and treated biofilms were analyzed for the biomass production, after 48 h of incubation at 37 °C on polystyrene surface, through Cristal Violet staining method. The results were expressed as average of OD595 values of three experiments (mean value ± SD). Symbol represents result statistically significant (p ˂ 0.05). Down; representative images of the in vitro mature biofilms at 37 °C on polystyrene surface untreated (a) and treated with LH IDROXI FAST (b) and LH ENZYCLEAN SPRAY (c). Biofilms were cultured for 48 h, stained with live/dead reagents, and visualized with the optical microscope fluorescence. Sessile population in biofilms stained in red (propidium iodide) expresses a compromised membrane integrity (damaged), whereas green stained bacteria (SYTO 9) remained viable. Both biocides reduced significantly S. aureus ATCC 6538 biomasses even exerting a killing effect. Original magnification ×1000. (ZIP 114 kb) [file 12199_2017_670_MOESM1_ESM.zip › Figure S1.jpg]
